# Multinucleated Giant Cells Induced by a Silk Fibroin Construct Express Proinflammatory Agents: An Immunohistological Study

**DOI:** 10.3390/ma14144038

**Published:** 2021-07-19

**Authors:** Sarah Al-Maawi, Xuejiu Wang, Robert Sader, Werner Götz, Antonella Motta, Claudio Migliaresi, Charles James Kirkpatrick, Shahram Ghanaati

**Affiliations:** 1 Department for Oral, Cranio-Maxillofacial and Facial Plastic Surgery, FORM (Frankfurt Orofacial Regenerative Medicine) Lab, University Hospital Frankfurt Goethe University, Theodor-Stern-Kai 7, 60590 Frankfurt am Main, Germany; sarah.Al-Maawi@kgu.de (S.A.-M.); R.Sader@em.uni-frankfurt.de (R.S.); kirkpatrick@uni-mainz.de (C.J.K.); 2 Department of Oral and Maxillofacial Surgery, Capital Medical University School of Stomatology, Beijing 100050, China; wangxuejiu@126.com; 3 Center of Dento-Maxillo-Facial Medicine, Department of Orthodontics, University of Bonn, Welschnonnenstrasse 17, 53111 Bonn, Germany; wgoetz@uni-bonn.de; 4 Biotech Research Center and INSTM Unit, Department of Industrial Engineering, University of Trento, 38123 Trento, Italy; Antonella.motta@unitn.it (A.M.); claudio.migliaresi@unitn.it (C.M.)

**Keywords:** silk fibroin, multinucleated giant cells, inflammation, disintegration, cellular response

## Abstract

Multinucleated giant cells (MNGCs) are frequently observed in the implantation areas of different biomaterials. The main aim of the present study was to analyze the long-term polarization pattern of the pro- and anti-inflammatory phenotypes of macrophages and MNGCs for 180 days to better understand their role in the success or failure of biomaterials. For this purpose, silk fibroin (SF) was implanted in a subcutaneous implantation model of Wistar rats as a model for biomaterial-induced MNGCs. A sham operation was used as a control for physiological wound healing. The expression of different inflammatory markers (proinflammatory M1: CCR-7, iNos; anti-inflammatory M2: CD-206, CD-163) and tartrate-resistant acid phosphatase (TRAP) and CD-68 were identified using immunohistochemical staining. The results showed significantly higher numbers of macrophages and MNGCs within the implantation bed of SF-expressed M1 markers, compared to M2 markers. Interestingly, the expression of proinflammatory markers was sustained over the long observation period of 180 days. By contrast, the control group showed a peak of M1 macrophages only on day 3. Thereafter, the inflammatory pattern shifted to M2 macrophages. No MNGCs were observed in the control group. To the best of our knowledge, this is study is the first to outline the persistence of pro-inflammatory MNGCs within the implantation bed of SF and to describe their long-term kinetics over 180 days. Clinically, these results are highly relevant to understand the role of biomaterial-induced MNGCs in the long term. These findings suggest that tailored physicochemical properties may be a key to avoiding extensive inflammatory reactions and achieving clinical success. Therefore, further research is needed to elucidate the correlation between proinflammatory MNGCs and the physicochemical characteristics of the implanted biomaterial.

## 1. Introduction

Currently, a wide variety of biomaterials are available for clinical applications in different fields to support both soft and bone tissue regeneration [1,2]. In addition to suitable biomechanical properties, biomaterials must meet different biological and clinical requirements [3]. In particular, biodegradable and natural polymers aim to restore damaged tissue and integrate into the recipient area. During the biomaterial resorption process, high inflammation and adverse reactions may demand revision surgery or cause failure of augmentation. In the past decade, different natural polymers have been widely investigated and developed for use as biomaterials, including collagen [4], chitosan [5], alginate [6], and silk [1]. Their large availability and biocompatibility make them an important source of materials that can be applied to replace damaged tissue in many fields. Specifically, silk fibroin (SF), extracted from *Bombyx mori,* showed promising results in different studies. SF particles and lyophilized SF were shown to be sufficient for loading with active molecules as a drug delivery device [7,8]. Other studies implemented SF in combination with collagen to develop a 3D bioprintable material with reinforced mechanical properties [9]. Additionally, SF was implanted in mice and showed successful results when utilized as an arterial vascular graft [10]. Another in vivo study analyzed the application of SF in combination with hyaluronic acid and zinc oxide for the treatment of burn injuries and showed improved wound healing and reduced inflammatory reactions [11].

After material implantation, a complex interplay takes place between the biomaterial and the host tissue [12]. This complex biomaterial-based regeneration process is still poorly understood. However, material physicochemical properties such as roughness, porosity, hydrophilicity, and polarity primarily influence the cellular reaction and immune responses to implanted biomaterial [13]. Many studies have shown that the biomaterial-induced cellular reaction determines regenerative capacity and thereby clinical outcomes [14,15,16]. Macrophages and lymphocytes, as part of the immune system, play a crucial role in wound healing and the biomaterial-based regeneration process [17,18]. After macrophages reach and adhere to the biomaterial, they undergo activation and differentiation [19]. The so-called classical activation leads to the differentiation of macrophages into the proinflammatory type (M1) [20]. They release different inflammatory signaling molecules, such as C-C chemokine receptor type 7 (CCR7), and chemokines, such as CCL2, CCL4, CXCL8, and inducible nitric oxide synthase (iNOS) [21]. M1 macrophages were shown to have enhanced degradative potential and release degrading enzymes such as reactive oxygen species [21]. Similar to their role in the wound healing process, biomaterial-adherent macrophages can eventually differentiate through alternative activation into the anti-inflammatory type (M2) [21]. M2 macrophages express proteins such as CD-206 and CD-163. and release anti-inflammatory cytokines such as interleukin 10 [21]. They exhibit a reduced degradative capacity and contribute to tissue repair and remodeling by recruiting regenerative cells to the region [21]. The plasticity of macrophages allows them to shift from one type to another according to their microenvironment and in response to the implanted biomaterial [22].

In addition to macrophages, multinucleated giant cells (MNGCs) are frequently observed in the implantation bed of different biomaterials [23]. Various studies analyzed their formation process in response to biomaterials. MNGCs form as a result of macrophage fusion on the biomaterial surface after they fail to degrade the biomaterial in a so-called process of frustrated phagocytosis [24]. This formation may reflect their attempt to recognize the material as a foreign body and consequently leads to material encapsulation or degradation. Interestingly, the pathophysiology and role of biomaterial-induced MNGCs in the degradation process of biomaterials a are still unexplored. Notably, in vivo studies have shown that the number of induced MNGCs is primarily related to biomaterial-specific physicochemical properties [25]. For example, changes in the size and morphology of beta tricalcium phosphate granules led to significant changes in the number of induced MNGCs in vivo [26,27]. In a previous study, we have shown that silk fibroin treated with formic acid for 60 min induced a higher number of MNGCs compared to SF treated with formic acid for 30 min [28]. However, the role of MNGCs was still unanalyzed. Thereby, the present follow-up study aimed to analyze the inflammatory pattern and differentiation of silk fibroin-induced macrophages and MNGCs in vivo over a period of 180 days. Special interest was directed to the analysis of the expression kinetics of macrophages and MNGCs over 180 days to understand better their role in the success or failure of biomaterials.

## 2. Materials and Methods

This study is a retrospective follow-up analysis of paraffin blocks available from a previous animal study [28] that aimed to analyze the inflammatory pattern of silk fibroin-induced macrophages and multinucleated giant cells over a long period of 180 days.

### 2.1. Three-Dimensional Nonwoven Silk Fibroin Scaffolds

The detailed production process of SF 3D nonwoven scaffolds is described in our previous study [28,29]. Briefly, *B. mori* cocoons were degummed by boiling to remove sericin. Next, the degummed silk was washed in distilled water and dried at room temperature. The 3D nonwoven substrates were prepared according to the method previously described by Armato et al. [30] The fiber suspension was then shaken in a formic acid (98%) solution at room temperature for 60 min. After formic acid evaporation under atmospheric conditions, the resulting nonwoven silk fibroin was washed several times with double-distilled water and dried under vacuum, resulting in 3D nonwoven micronets. The biomaterial was sterilized via gamma irradiation (25 kG).

### 2.2. Animal Experiments

**Figure 1:**
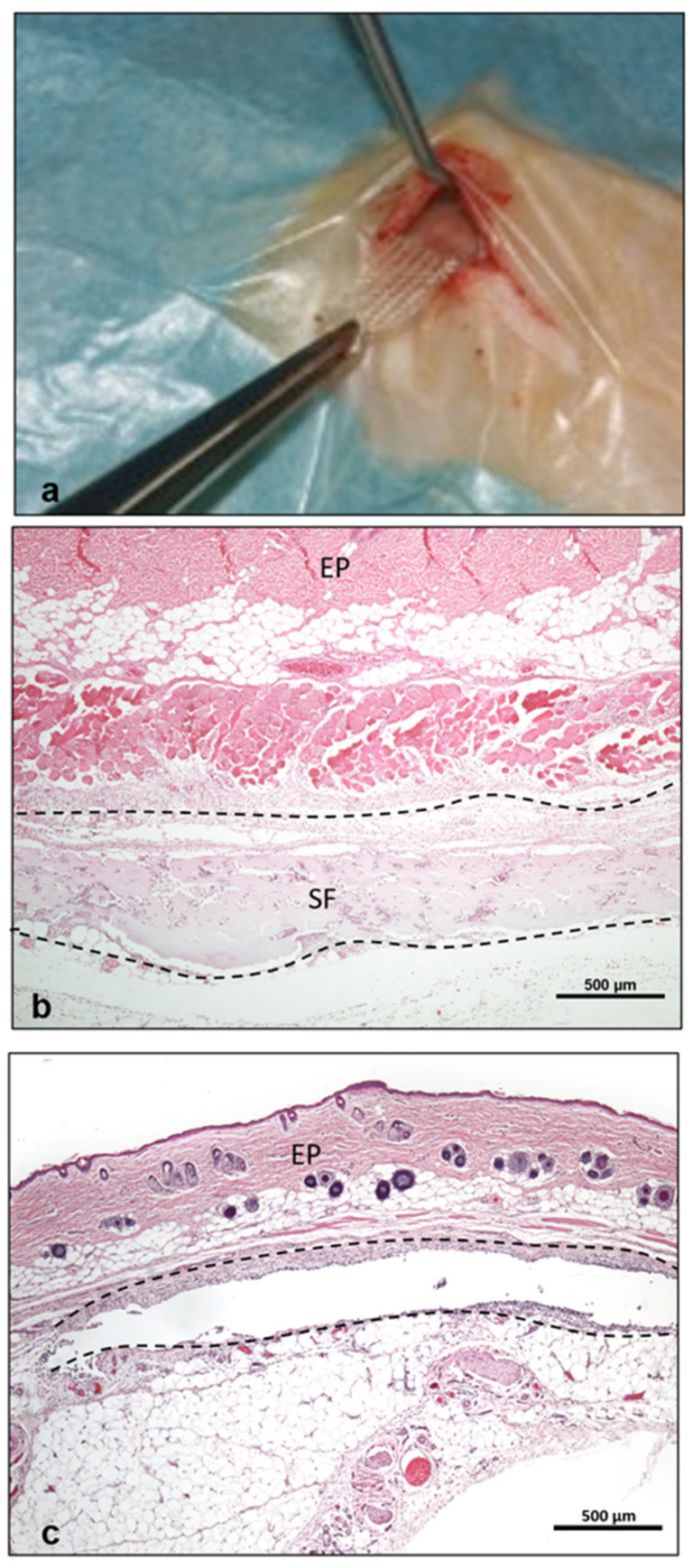
Representative images of (**a**) silk fibroin implantation in the subcutaneous model. The skin tissue is presented histologically showing the epithelium (EP) and the subcutaneous pocket on day 3. (**b**) shows the test group with the wound area (dashed line) including the implanted biomaterial (SF) and (**c**) shows the sham operated control group including the wound area (dashed line). Histological pictures are presented with hematoxylin-eosin staining at 40× magnification. Scale bar = 500 µm.

This study was approved by the Committee on the Use of Live Animals in Teaching and Research (Landesuntersuchungsamt) of the State of Rhineland Palatinate, Germany (AZ:23177-07/G06-1-016) as previously published [28]. Silk fibroin was implanted in the subcutaneous model of 5-week-old Wistar rats (Charles River Laboratories, Sulzfeld, Germany) as previously described [28] (Figure 1b). Additionally, sham-operated animals were used as a control group [28]. A sham operation is a control surgical intervention that includes all surgical steps that were performed for the test group, but without implantation of a biomaterial. The sham operation therefore mimics physiological wound healing without silk fibroin (Figure 1a). The implantation area within the peri-implantation region was explanted after 3, 10, 15, 30, 60, 90, 120 and 180 days and fixed in histofix for 24 h before further histological processing.

### 2.3. Histological and Immunohistological Staining

Histological processing and sectioning were performed as previously described [31,32]. Seven paraffin slices of 3–4 µm thickness were cut using a rotation microtome (Leica RM2255, Wetzlar, Germany). Samples of four individual animals were used for each group and time points (*n* = 4 for each group and time point). From each sample, the first slide was used for hematoxylin and eosin staining. The second slide was used for tartrate-resistant acid phosphatase (TRAP) staining, and the next five slides were used for immunohistochemical staining, as previously described [31]. In brief, after deparaffinization and rehydration using xylol and an alcohol series of decreasing concentrations, the slides were pretreated according to the respective antibody (Table 1) and processed serially (UltraVision^TM^ Quanto Detection System, ThermoFisher Scientific, Germany) with avidin and biotin blocking solutions (Avidin/Biotin Blocking Kit, Vector Laboratories, USA). Subsequently, the slides were washed with 10% normal goat serum in 1% BSA in TBS buffer. Next, the primary antibody was applied (Table 1). After the respective incubation time, the slides were then incubated in the secondary antibody (goat anti-rabbit IgG-B, sc-2040, 1:200, Santa Cruz Biotechnology, USA) for 30 min at room temperature followed by an ABC (ThermoFisher Scientific, Germany) composite incubation for another 30 min. AEC chromogen (ThermoFisher Scientific, Germany) was then applied to reveal the target antigens. Finally, the samples were counterstained with hematoxylin for 10 min followed by blueing for 5 min.

### 2.4. Histomorphometric Analysis

Histomorphometric analysis was performed using NIS-Elements (Nikon Tokyo, Japan) software, using the “count” tool according to the manufacturer’s instructions. Briefly, images were obtained with a DS-Fi1/digital camera connected to an Eclipse 80i histological microscope (Nikon). Immunohistochemically or TRAP-stained slides were used (*n* = 4 per group and time point) to calculate the number of positively stained target cells. Five (×200 magnification) randomly selected fields of one slide were used to count the cell numbers. The final number of the target cells was the mean value of the five fields, and the final result was calculated as the cell number/mm^2^.

### 2.5. Statistical Analysis

The calculated data (*n* = 4 per time point and group) are expressed as the mean ± the standard deviation. Statistical analysis was performed using GraphPad Prism 7.0 software (GraphPad Software Inc., La Jolla, CA, USA) by one-way and two-way analysis of variance (ANOVA) with Tukey’s multiple comparisons test (α = 0.05). Differences were considered statistically significant if the *p* values were * *p* < 0.05, and highly significant if the *p* values were, ** *p* < 0.01, *** *p* < 0.001, and **** *p* < 0.0001.

## 3. Results

### 3.1. Qualitative Analysis

#### 3.1.1. Control Group

**Figure 2:**
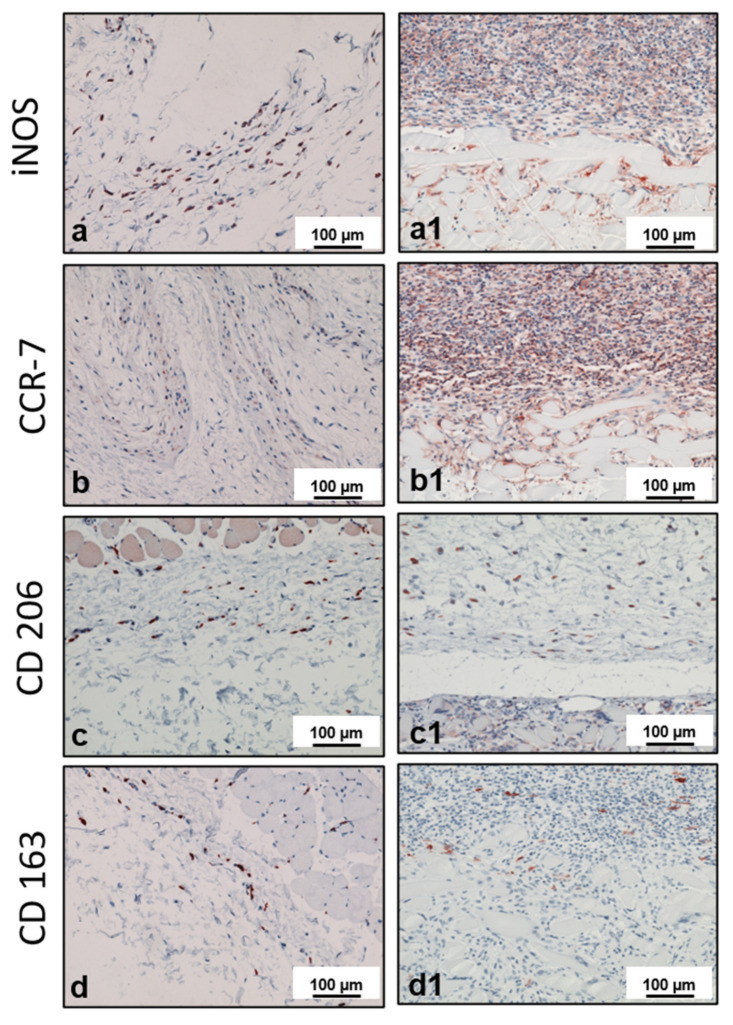
Macrophage polarization on day 15. Positively stained cells are depicted in a brown-red color. (**a**–**d**) Expression of the different markers in the control group. Panels (**a1**–**d1**) show the expression of different markers in the silk fibroin group. All pictures taken at 200× magnification. Scale bars = 100 µm.

The animals in the control group received sham operations without silk fibroin to evaluate the physiological wound healing pattern. After 3 days, an accumulation of inflammatory cells was observed. Most of the cells expressed the proinflammatory markers iNOS and CCR-7, and few mononuclear cells (MNCs) expressed the anti-inflammatory markers CD-163 and CD-206. On day 10, a shift in the inflammatory pattern of the MNCs was observed. Thereby, CD-206 was higher expressed than all other markers evaluated. At this time point, new vessel formation was detected within the operation region. Around day 15, the number of inflammatory cells was markedly reduced. Few cells were positive for iNOS, CCR-7, CD-206, or CD-163 (Figure 2a–d). On day 30, the wound area exhibited total regeneration. No MNC accumulation was found in the operation region. Some iNOS (+), CCR-7 (+), CD-206 (+), and CD-163 (+) cells were sporadically distributed in the evaluated tissue. Similar results were observed from day 60 to 180 without observable changes. No multinucleated giant cells (MNGCs) were found at any time point.

#### 3.1.2. Silk Fibroin Group

**Figure 3:**
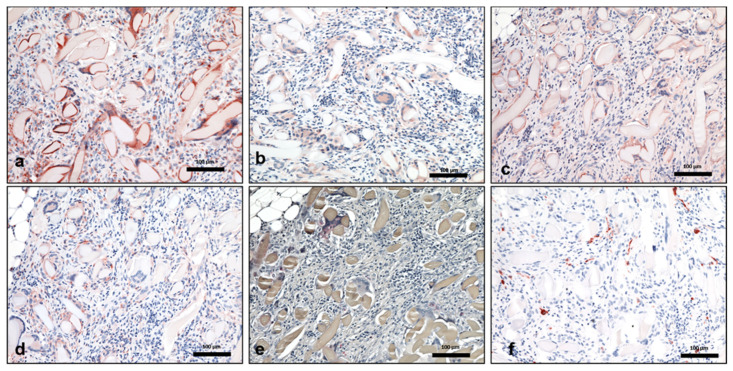
Multinucleated giant cell polarization using immunohistochemical staining on day 15. Positively stained cells are depicted in a brown-red color. (**a**) CD-68 (+) MNGCs. (**b**) iNOS (+) MNGCs. (**c**) CCR-7 (+) MNGCs. (**d**) CD-206 (+) MNGCs. (**e**) Some TRAP (+) MNGCs. (**f**) CD-163-negative MNGCs. All pictures are captured at 200× magnification. Scale bars = 100 µm.

**Figure 4:**
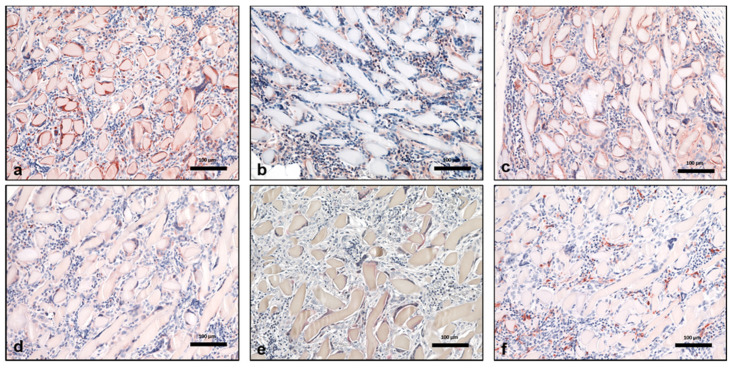
Multinucleated giant cell polarization using immunohistochemical staining on day 60. Positively stained cells are stained in a brown-red color. (**a**) CD-68 (+) MNGCs. (**b**) iNOS (+) MNGCs. (**c**) CCR-7 (+) MNGCs. (**d**) CD-206 (+) MNGCs. (**e**) TRAP (+) MNGCs. (**f**) CD-163-negative MNGCs. All pictures are taken at 200x magnification. Scale bars = 100 µm.

**Figure 5:**
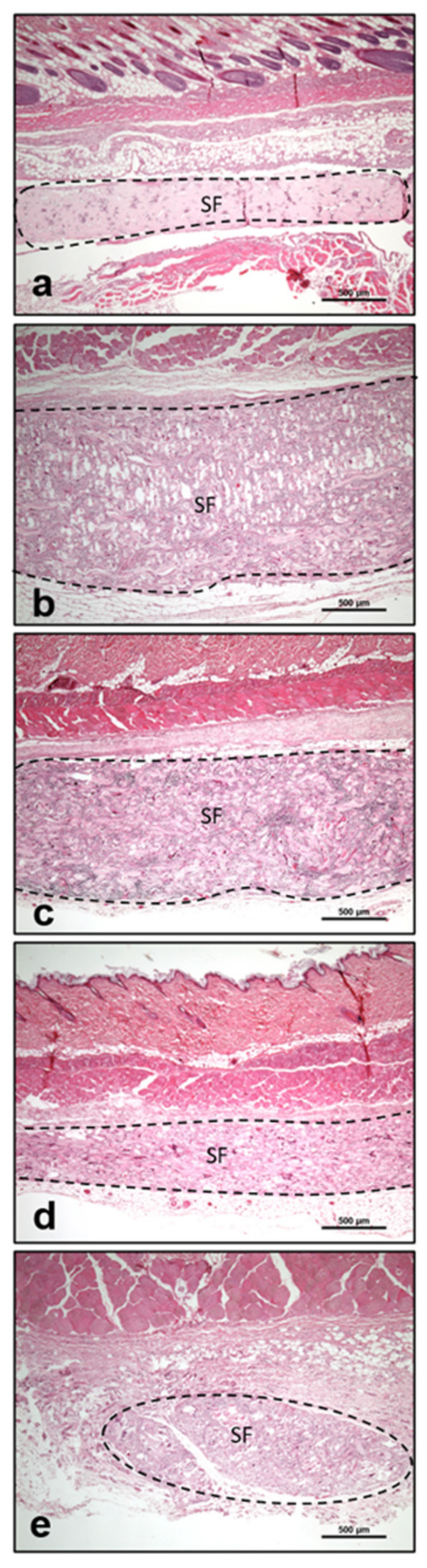
The disintegration process of silk fibroin (SF) after the induction of MNGCs. (**a**) SF on day 3. (**b**) SF on day 15 with a high number of MNGCs penetrating the biomaterial. (**c**) SF on day 60 with a markedly reduced fiber diameter and significant ingrowth of connective tissue. (**d**) SF on day 90, completely invaded by MNGCs and connective tissue. (**e**) Remnants of SF on day 180 with a high number of MNGCs and connective tissue invasion. All pictures are presented in hematoxylin-eosin staining, at 40× magnification. Scale bar = 500 µm.

Three days after implantation, the material was infiltrated by a few MNCs. Most of them expressed the proinflammatory markers iNOS and CCR-7. On day 10, the number of MNCs increased markedly. More cells were detected towards the center region of the material. At this time point, a high number of MNCs expressed the anti-inflammatory marker CD-206 in addition to other MNCs that were positive for the markers iNOS, CCR-7, and CD-163. Interestingly, at this time point, MNGCs were observed on the fiber surfaces. Additionally, new vessel formation was observed in the peripheral parts of the biomaterial. Most of the MNGCs expressed CD-68, iNOS, and CCR-7. Only a few cells expressed CD-206 and TRAP. Near day 15, both the numbers of MNCs and MNGCs increased in comparison to the previous time point. The material was embedded in a cell-rich connective tissue that invaded the biomaterial, including its central region. MNGCs were also observed in the central part of the material. The fibers showed signs of degradation, and the material started to lose its initial fibrous structure. At this time point, the MNCs expressed inflammatory signaling molecules (iNOS and CCR-7) rather than anti-inflammatory signaling molecules (CD-206 and CD163) (Figure 2a1–d1). A similar differentiation pattern was observed for the MNGCs at this time point (Figure 3a–f). Thirty days after implantation, the number of MNCs was reduced in comparison to the previous time points. Additionally, CCR-7 and CD-206 were the most highly expressed markers of MNCs at this time point. In contrast, the number of MNGCs increased markedly. Here, most of the MNGCs expressed CD-68, iNOS, and CCR-7. Moreover, the vessels were distributed throughout the biomaterial. From day 60 to 180, the number of MNCs appeared stable. Similarly, most of the MNCs expressed iNOS, CCR-7, and CD-206. However, very few cells were positive for CD-163. Interestingly, the number of MNGCs increased markedly from day 30 to 180. Most of the MNGCs contained CD-68, iNOS, and CCR-7 throughout these evaluation time points (Figure 4a–f). Over the observation period, SF underwent premature degradation starting on day 15 by losing its initial structure and allowing the connective tissue and inflammatory cells to completely invade its central region. This disintegration pattern continued until day 180, at which time only some material remnants were found (Figure 5a–e).

### 3.2. Quantitative Analysis

#### 3.2.1. Differentiation of Macrophages over the Evaluation Time Points

**Figure 6:**
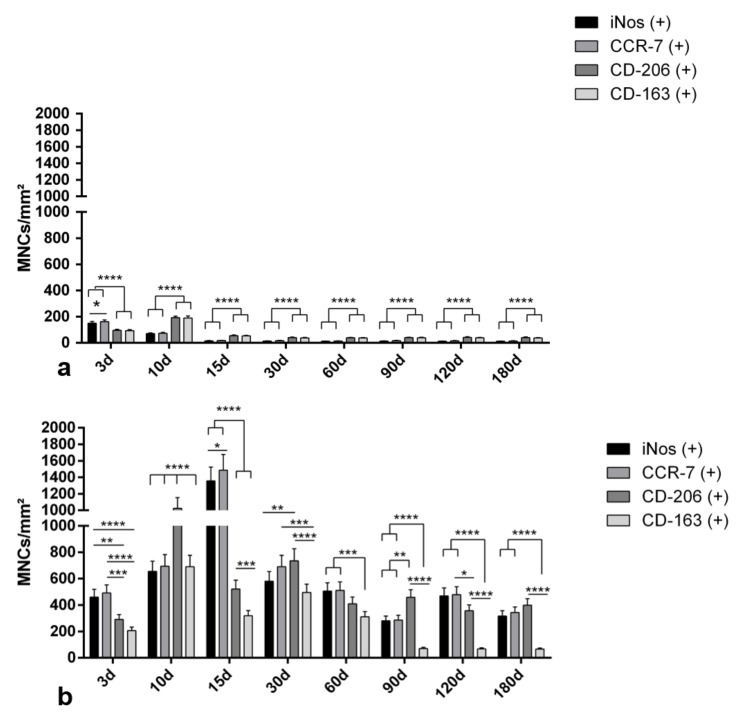
Histomorphometric analysis of the differently polarized macrophages of (**a**) the control group and (**b**) the silk fibroin group. Differences were considered statistically significant if the *p* values were * *p* < 0.05 and highly significant if the *p* values were, ** *p* < 0.01, *** *p* < 0.001, or **** *p* < 0.0001; d = days.

In the control group, the early evaluation time point (day 3) exhibited a number of proinflammatory cells CCR-7 (+) and iNOS (+), that were significantly higher compared to the number of the CD-206 (+) and CD-163 (+) MNCs (**** *p* < 0.0001 for both). However, starting on day 10, most of the MNCs shifted to the anti-inflammatory polarization. This pattern continued until the end of the study on day 180. At the time points of evaluation within the period 10–180 days (10, 15, 30, 60, 90, 120, and 180 days), the numbers of anti-inflammatory CD-206 (+) and CD-163 (+) MNCs were significantly higher than those of the proinflammatory CCR-7 (+) and iNOS (+) MNCs (**** *p* < 0.0001 for all mentioned time points) (Figure 6a).

In the test group (SF), a different inflammatory pattern was observed. In the early time point of 3 days, the implantation region was mostly dominated by the proinflammatory MNCs iNos (+), CCR7 (+). Their number was significantly higher compared to the anti-inflammatory MNCs (for example ** *p* < 0.01 for CCR7 (+) vs. CD-206 (+) and *** *p* < 0.001 for CCR7 (+) vs. CD163 (+)). However, with the progress of time, the number of CD-206 (+) cells increased markedly, especially between day 10 and 15 after implantation. It was even significantly higher compared to the proinflammatory cells iNos (+) and CCR-7 (+). From day 30 onwards, the inflammatory pattern was dominated firstly by the proinflammatory cells (CCR7 (+) and iNos (+)) and secondly by the anti-inflammatory CD-206. However, the expression of a further anti-inflammatory surface marker, CD-163 (+), was continuously significantly lower than for all other markers for the time points at 90, 120, and 180 days (Figure 6b).

#### 3.2.2. Kinetics of Macrophage Polarization over the Evaluation Time Period

**Figure 7:**
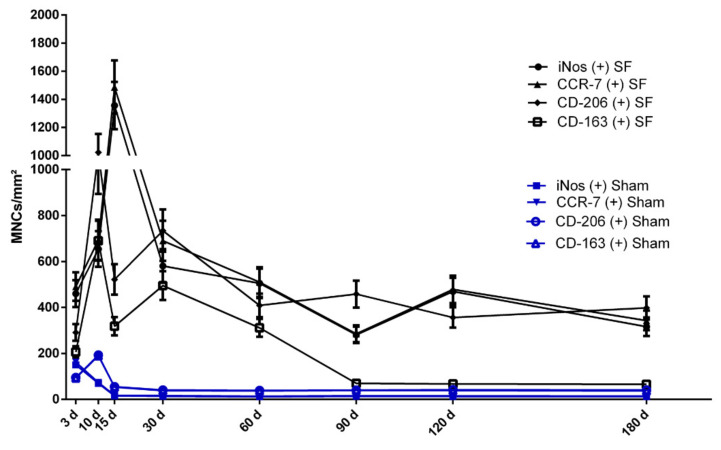
Histomorphometric analysis of the polarization kinetics of macrophages over the total observation time period highlights the differences between the control group and the silk fibroin group. statistical analysis is presented in Table S1; d = days.

In the control group, which represents physiological wound healing, the numbers of proinflammatory MNCs (iNOS (+) and CCR-7 (+) cells) showed a peak on day 3, and their numbers progressively decreased through day 10 to reach their minimum on day 15. This level persisted until day 180. In contrast, the numbers of anti-inflammatory MNCs (CD-206 (+) and CD-163 (+)) increased from day 3 to day 10 to reach their peak. However, around day 15, their numbers showed a strong reduction but were still higher than the number of the proinflammatory MNCs iNOS (+) and CCR-7 (+). This level continued until the end of the study on day 180 (Figure 6). A different pattern was observed in the SF group. In this case, the number of the proinflammatory MNCs (iNOS (+) and CCR-7 (+) cells) was higher than that of the anti-inflammatory MNCs (CD-206 (+) and CD-163 (+) cells) at almost all time points. Proinflammatory MNCs (iNOS (+) and CCR-7 (+) cells) showed the highest number on day 15. Near day 30, their number was highly reduced, but was still higher than that of anti-inflammatory MNCs. This pattern continued until the end of the study on day 180. Interestingly, the first peak of the anti-inflammatory MNCs was reached by CD-206 (+) MNCs on day 10; however, their numbers decreased around day 15. A similar pattern was detected for the CD-163 (+) MNCs. Their number was dramatically reduced from day 30 until the end of the study on day 180. These kinetics showed that in the SF group, more MNCs were recruited, especially pro-inflammatory MNCs, over the total study period (Figure 7, Table S1).

#### 3.2.3. Differentiation of Multinucleated Giant Cells over the Evaluation Time Period

**Figure 8:**
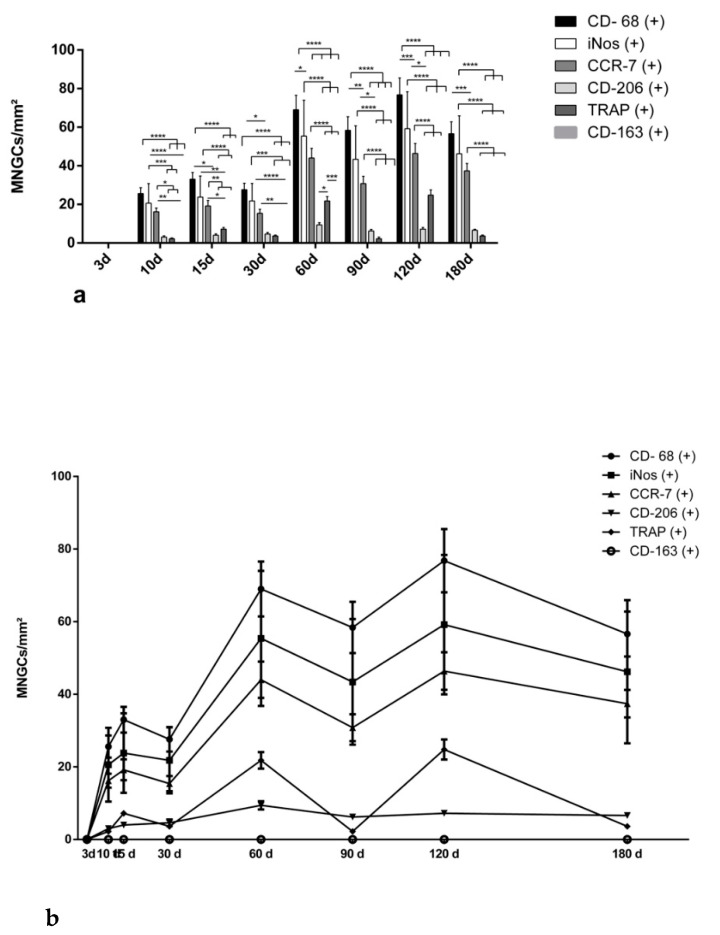
Histomorphometric analysis of the polarization of multinucleated giant cells in the silk fibroin group. (**a**) Statistical analysis to compare the numbers of pro- and anti-inflammatory MNGCs at each time point. Differences were considered statistically significant if the *p* values were * *p* < 0.05 and highly significant if the *p* values were, ** *p* < 0.01, *** *p* < 0.001 or **** *p* < 0.0001. (**b**) Polarization kinetics of MNGCs over the observation time period. Statistically significant differences are not presented in this figure; d = days.

No multinucleated giant cells (MNGCs) were found in the control group at any time point. In the group of SF, MNGCs were evident, starting with day 10. At that timepoint, most of them expressed CD-68 (+) and proinflammatory markers (iNOs (+) and CCR-7 (+)). The number of proinflammatory MNGCs was significantly higher compared to those that expressed anti-inflammatory markers such as CD-206 (+) and CD-163 (+) (for example: *** *p* < 0.001 for iNos (+) vs. CD-206 (+)). This pattern continued until the end of the study on day 180. Interestingly, the number of TRAP (+) MNGCs was the highest within the time period between day 60 and 120. It was significantly higher than the number of MNGCs expressing anti-inflammatory markers (for example day 60: * *p* < 0.05 for TRAP (+) vs. CD-206 (+)). At the last time point of the study (day 180), the number of proinflammatory MNGCs (CCR-7 (+), iNos (+)) was sustained at a high level and was significantly higher compared to the number of anti-inflammatory MNGCs (for example *** *p* < 0.0001 for CCR-7 (+) vs. CD-206 (+) and CD-163 (+), (Figure 8a).

#### 3.2.4. Kinetics of Multinucleated Giant Cell Polarization over the Evaluation Time Period

The numbers of the differently polarized MNGCs showed increasing kinetics for almost all evaluated markers. CD-68 (+) MNGCs were the most abundant at all time points. Their numbers increased from day 10 to 120 continuously, and then showed a slight decrease around day 180. A similar pattern was observed for the iNOS (+) and CCR-7 (+) MNGCs. TRAP (+) MNGC numbers were lower than those of the previously described markers. Interestingly, TRAP (+) MNGCs showed two peaks at days 60 and 90 after implantation, but were reduced dramatically near day 180. The numbers of the CD-206 (+) MNGCs were lower than those of the CD-68 (+) MNGCs and MNGCs with the pro-inflammatory markers iNOS and CCR-7 at all time points. They showed a slight increase from day 10 to 30 after implantation and maintained a constant number from day 60 to the end of the study on day 180. No CD-163 (+) MNGCs were found at any time point (Figure 8b).

## 4. Discussion

Biomaterials are widely used to support wound healing and regeneration in different fields of medicine [33,34]. According to the properties of the implanted biomaterial, the cellular reactions and molecular expression patterns of the wound may be influenced [13,35,36]. The present immunohistochemical study in a subcutaneous implantation model analyzed cellular reactions and inflammatory patterns in response to degummed silk fibers treated in formic acid for 60 min (SF), compared to physiological wound healing. The main aim was to analyze the long term polarization pattern of macrophages and SF-induced multinucleated giant cells (MNGCs) in pro- and anti-inflammatory phenotypes for 180 days to better understand their role in the success or failure of biomaterials.

Generally, biomaterials are implanted in a surgically created wound [37]. Therefore, the SF-induced cellular reaction interferes with wound healing in a complex regenerative process. The biomaterial first contacts the host tissue via blood [38]. Thereafter, cells of the immune system, such as macrophages and lymphocytes, are attracted to the implantation site [39]. Macrophages exhibit different adhesion molecules that allow them to attach to the biomaterial surface and become activated accordingly [40]. Interestingly, the results of the present study showed that macrophages in the control group expressed pro-inflammatory molecules such as iNOS and CCR-7 mainly in the early wound healing phase (3–10 days). However, their number was reduced markedly near day 15. At this time point, anti-inflammatory macrophages (CD-206, CD-163 (+)) predominated as a sign of continuing wound healing. After 30 days, both macrophage phenotypes (M1 and M2) maintained a low level until the end of the study on day 180 (Figure 6). This reaction represents the phases of the physiological wound healing process, which involves mainly M1 macrophages in the first phase of acute inflammation [41]. In contrast, the SF group showed a significantly higher number of M1 macrophages (iNOS (+), CCR-7 (+)) starting on day 3 (Figure 6). Their number was reduced near day 30 but statistically significantly higher numbers were maintained compared to the numbers observed in the control group. Additionally, the number of proinflammatory M1 macrophages dominated this process over the total study time. The persistence of macrophages in the SF group, especially M1 (iNOS (+) and CCR-7 (+)), represents the changes from physiological wound healing to chronic inflammation induced by the implanted biomaterial [37,39]. In this context, the surface topography and properties of the biomaterial directly influenced the macrophage reaction [42]. Specifically, the here-evaluated SF construct was treated with formic acid for 60 min and was shown to exhibit a less porous structure compared to SF constructs treated with formic acid for 30 min [28]. Additionally, the extensive treatment with formic acid enhanced the stability of the membrane, as demonstrated earlier [28]. In this context, it was shown that the degradation rate of SF constructs treated for 60 min revealed delayed the immune response when compared to SF constructs treated for 30 min [28]. Thus, SF constructs treated for 60 min persisted for 90 days before undergoing degradation. The present investigation also analyzed the expression pattern of SF-induced cells. The results showed that the number of proinflammatory macrophages reached a peak on day 15 and decreased thereafter towards day 180. At the same time the multinucleated giant cells first appeared around day 15 and expressed proinflammatory agents continuously up to day 180. One reason may be the persistence of the here evaluated SF construct and its resistance to degradation, which led to the formation of MNGCs as a foreign body reaction [28]. However, the present retrospective study failed to determine a specific parameter that led to the here observed reaction. Due to sample limitation, a thorough characterization of the physicochemical characteristics of the analyzed SF constructs was not possible. Nevertheless, the presented findings again highlight that the physicochemical composition and properties of the biomaterial can be influenced using a various of chemical and physical methods. Most importantly is to recognize that alterations in the biomaterial surface and composition may directly influence the cellular reaction. Previous studies have shown that hydrophilic and anionic surfaces reduce macrophage adhesion [43]. Additionally, material shapes with sharp edges, such as triangular or pentagon-like shaped particles, enhance inflammation in comparison to more rounded materials [21,37,44]. Similarly, the modification of silk fibroin by formic acid treatment was shown to change its biomechanical structure and reduce its degradation in vivo [28]. A further recent study showed that the application of mechanical pressure on a collagen matrix leads to a reduction in its porosity, thereby resulting in a higher inflammatory response. In that study, a significantly higher number of proinflammatory macrophages was found in the compressed collagen scaffold compared to the number of proinflammatory macrophages in the non-compressed porous scaffold [45].

In addition to the chronic inflammation induced by the persistent macrophages detected in this study, MNGCs were observed in the SF group, starting on day 10. In contrast, no MNGCs were revealed in the control group at any time point. This observation emphasizes that MNGCs are a sign of a foreign body reaction and are not part of physiological wound healing (Figure 7). Notably, during the formation period of MNGCs between days 10 and 15, the number of macrophages decreased dramatically from day 10/15 to day 30 (Figure 6). During the same time, the number of MNGCs increased continuously. Similar phenomena were also previously observed during MNGC formation in the implantation beds of different collagen matrices [4,46]. The reduction in the macrophage number may be a physiological process present in wound healing over time. Another reason for the reduction in macrophage number may be related to their fusion to form MNGCs [47]. Macrophages were shown to be precursor cells of MNGCs [3]. However, to date, little is known about the formation process and function of MNGCs. It has been reported that the fusion of macrophages into MNGCs may be a technique to avoid apoptosis and enhance the phagocytosis capacity as a response to foreign bodies [35,48].

Only a few studies have focused on the relationship between macrophage activation and the formation of MNGCs. In vitro studies showed that the treatment of monocytes with interleukin 3 and 14 leads to their fusion and the formation of MNGCs [49]. Further studies have shown that the inability of macrophages to phagocytose the biomaterial leads to a high inflammatory response in the context of frustrated phagocytosis [50]. In this process, the size and shape of the phagocytosed or internalized particle directly influences the inflammatory response of the cells, i.e., macrophages [50]. Another study showed that the signaling receptor dectin-1 plays an important role in the process of internalization. Therefore, particles that are too large and cannot be internalized by macrophages result in elevated levels of proinflammatory cytokines [51]. Moreover, this response is further enhanced by the longer exposure of the cells to the large particles [51]. These findings are in agreement with the results of the present study, showing a prolonged and persistent proinflammatory response of macrophages induced by the implanted SF, this being followed by the formation of MNGCs. Consequently, some studies described MNGCs as specialized phagocytes that degrade larger particles [52]. However, both M1 and M2 macrophages were found to be involved in the foreign body response process [39]. Specifically, adhesive molecules such as integrin beta 3 were found to be mandatory for the formation of MNGCs [53]. An in vivo study showed that CC chemokines such as CC chemokine ligand 2 (CCL-2) are also involved the formation of MNGCs. When implanting the alginate-based biomaterial in CCL-2 null mice and wild-type mice, CCL-2 null mice formed a significantly lower number of MNGCs around the biomaterial compared to wild-type mice. Additionally, a lower number of MNGCs was accompanied by the presence of more new collagenous tissue formation [54]. Another study showed that specific inflammatory molecules, such as CCL-2, were highly expressed by foreign body MNGCs but not by osteoclasts [55].

To the best of our knowledge, no studies have yet evaluated the pro- and anti-inflammatory responses of silk fibroin constructs in induced MNGCs over a period of 180 days. The present study showed that the SF-induced MNGCs studied here express proinflammatory molecules such as iNOS and CCR-7 rather than anti-inflammatory molecules such as CD-206. Interestingly, most of the MNGCs expressed CD-68, which is a general marker of macrophages. This observation emphasizes that the MNGCs observed here are formed from their precursors, macrophages. Additionally, the number of proinflammatory MNGCs increased continuously from day 10 to 60, and maintained a rather high level until day 180. During the observation time period, no shift into the anti-inflammatory type was observed. However, some MNGCs expressed the mannose receptor CD-206, which is suggested to be an anti-inflammatory marker [56,57]. Previous in vitro studies showed similar results for MNGCs derived from monocytes. The authors suggest that the macrophage mannose receptor CD-206 may be a key player in the formation process of MNGCs. The expression of CD-206 by MNGCs may be a sign of their potential endocytic or phagocytotic function [58,59]. Likewise, a previous immunohistochemical study analyzed the expression pattern of SF-induced MNGCs in human biopsies of two different bone substitute materials six months after augmentation. The results showed that MNGCs induced by both synthetic and xenogeneic bone substitute materials expressed proinflammatory rather than anti-inflammatory molecules [31]. In this context, it was shown that none of the evaluated biomaterials were able to shift the MNGCs to induce anti-inflammatory molecules. Moreover, the inflammatory pattern of MNGCs was previously found to be associated with premature degradation of the biomaterial and a high level of connective tissue formation [31,60,61]. Additionally, MNGCs were observed in capsule formation around bone substitute materials as a final stage of the foreign body reaction [62]. The present results also showed that a low number of SF-induced MNGCs expressed TRAP. TRAP is a degradative enzyme that was first observed in osteoclasts and is thereby recognized as an osteoclast marker [63]. However, several studies have shown that biomaterial-induced MNGCs also have the potential to express TRAP [60,61,64,65]. Moreover, it is important to note that there are different types of TRAP enzymes. TRAP 5a is suggested to be expressed in relation to an inflammatory response [66]. Based on these data, TRAP expression may also be a sign of a proinflammatory response in SF-induced MNGCs.

Different studies have pointed out the importance of biomaterial-specific physicochemical characteristics essential for inducing the inflammatory response [12,13]. Based on these facts, a recent study suggested a new classification of biomaterials according to the induced cellular reactions and the formation kinetics of MNGCs by analyzing the cellular reactions of up to 29 different materials in a subcutaneous implantation model for 30 days [25]. The authors suggest three classes. Class I included biomaterials that did not induce any MNGCs at any time point [25]. Class II represented biomaterials that induce an initial number of MNGCs and maintain a sustained number over 30 days, while class III included biomaterials that induce a continuously increasing number of MNGCs over 30 days. Given the present results, SF meets the criteria of a class III material, as it induces a continuously increasing number of MNGCs over time [25].

In the present study, the formation of MNGCs in response to the degummed SF treated in formic acid for 60 min led to biomaterial disintegration (Figure 5). Therefore, the SF was still detectable throughout the study period but lost its initial structure beginning on day 30 after implantation. The process of biomaterial disintegration by MNGCs was previously observed for different polymeric materials that underwent premature breakdown by the induction of MNGCs [29,67]. By contrast, polymeric materials that do not induce MNGCs undergo integration into the host tissue and preserve their initial structure, functioning as scaffolds and place-holders [29]. However, it is important to point out that modifications of the SF processing and preparation protocol may lead to different inflammatory patterns according to the resulting physicochemical properties. Additionally, one limitation of the present study is the evaluation of only one biomaterial, as it could be postulated that different materials induce different inflammatory patterns. Therefore, further studies will have to explore whether changing the biomaterial properties may influence biomaterial-induced MNGC differentiation. Altogether, the present study highlighted the proinflammatory capacity of SF-induced MNGCs. These findings are clinically interesting, as biomaterial-induced MNGCs are frequently observed in the implantation beds around inserted materials. In this context, the question arises as to whether proinflammatory MNGCs are useful for the regeneration process or whether they should be considered as an adverse reaction following chronic inflammation. Therefore, further studies are needed to explore the function of SF-induced MNGCs in different microenvironments.

## 5. Conclusions

The present study analyzed the inflammatory pattern of degummed silk fibers treated in formic acid for 60 min (SF) after implantation in a subcutaneous implantation model in comparison to physiological wound healing (control group). The findings underline foremost the influence of proinflammatory macrophages and their persistence in the formation of MNGCs. Additionally, the long term inflammatory kinetics of MNGCs over a period of 180 days was described for the first time in this study. In this context, it was shown that MNGCs preferentially express proinflammatory signaling molecules. Unlike the macrophages in physiological wound healing, MNGCs do not undergo a polarization shift to express anti-inflammatory markers even after 180 days. These findings are of great interest in understanding the long-term role of biomaterial-induced MNGCs in the implantation bed of biomaterials.

## Figures and Tables

**Table 1:** The pretreatment details and applied antibodies for immunohistochemical staining.

Antibody	Dilution	Pretreatment
CD-68 (MCA341GA, Bio-Rad, CA, USA)	1:600	Citrate buffer(pH = 6.0)
iNOS (ab15323, Abcam, UK)	1:1000	Citrate buffer(pH = 6.0)
CCR-7 (ab32527, Abcam, UK)	1:2000	Citrate buffer(pH = 6.0)
CD-206 (ab64693, Abcam, UK)	1:2000	Citrate buffer(pH = 6.0)
CD-163 (bs-2527R, Bioss, MA, USA)	1:1000	EDTA buffer(pH = 9.0)

## Data Availability

Not applicable.

## Supplementary Materials

The following are available online at https://www.mdpi.com/article/10.3390/ma14144038/s1, Table S1: Statistical analysis comparing the differently polarized macrophages in the control group with the silk fibroin group at the different evaluation time points.
