# Supplementary material for: Multinucleated Giant Cells Induced by a Silk Fibroin Construct Express Proinflammatory Agents: An Immunohistological Study
Source: Materials (Basel). 2021 Jul 19;14(14):4038. doi: 10.3390/ma14144038 (PMC8307820; doi:10.3390/ma14144038)
Supplement: Supplementary file 1 [file materials-14-04038-s001.zip › materials-1176259-supplementary.pdf]

materials

Supplementary Materials: Multinucleated Giant Cells Induced by a Silk Fibroin Construct Express Proinflammatory Agents: An Immunohistological Study

Sarah Al-Maawi [1,†], Xuejiu Wang [2,†], Robert Sader [1], Werner Götz [3], Antonella Motta [4], Claudio Migliaresi [4], Charles James Kirkpatrick [1] and Shahram Ghanaati [1,*]

[1] Department for Oral, Cranio-Maxillofacial and Facial Plastic Surgery, FORM (Frankfurt Orofacial Regenerative Medicine) Lab, University Hospital Frankfurt Goethe University, Theodor-Stern-Kai 7, 60590 Frankfurt am Main, Germany; sarah.Al-Maawi@kgu.de (S.A.-M.); R.Sader@em.uni-frankfurt.de (R.S.); kirkpatrick@uni-mainz.de (C.J.K.)
[2] Department of Oral and Maxillofacial Surgery, Capital Medical University School of Stomatology, Beijing 100050, China; wangxuejiu@126.com
[3] Center of Dento-Maxillo-Facial Medicine, Department of Orthodontics, University of Bonn, Welschnonnenstrasse 17, 53111 Bonn, Germany; wgoetz@uni-bonn.de
[4] Biotech research center and INSTM Unit, Department of Industrial Engineering, University of Trento, 38123 Trento, Italy; Antonella.motta@unitn.it (A.M.); claudio.migliaresi@unitn.it (C.M.)
* Correspondence: Shahram.Ghanaati@kgu.de; Tel.: +49-69-6301-4492; Fax: +49-69-6301-3785
† Equal contribution.

Table S1: Statistical analysis comparing the differently polarized macrophages in the control group with the silk fibroin group at the different evaluation time points. Differences were considered statistically significant if the p values were *p< 0.05 and highly significant if the p values were, **p< 0.01, ***p< 0.001 or ****p<0.0001, n.s: not significant.

Time point	*iNOS*	*CCR-7*	*CD-206*	*CD-163*
3 days	**** p<0.0001	**** p<0.0001	**** p<0.0001	**** p<0.0001
10 days	**** p<0.0001	**** p<0.0001	**** p<0.0001	**** p<0.0001
15 days	**** p<0.0001	**** p<0.0001	**** p<0.0001	**** p<0.0001
30 days	**** p<0.0001	**** p<0.0001	**** p<0.0001	**** p<0.0001
60 days	**** p<0.0001	**** p<0.0001	**** p<0.0001	**** p<0.0001
90 days	**** p<0.0001	**** p<0.0001	**** p<0.0001	n.s.
120 days	**** p<0.0001	**** p<0.0001	**** p<0.0001	n.s.
180 days	**** p<0.0001	**** p<0.0001	**** p<0.0001	n.s.
